# Shikonin potentiates the effect of arsenic trioxide against human hepatocellular carcinoma *in vitro* and *in vivo*

**DOI:** 10.18632/oncotarget.12041

**Published:** 2016-09-15

**Authors:** Jingjing Song, Zhongwei Zhao, Xiaoxi Fan, Minjiang Chen, Xingyao Cheng, Dengke Zhang, Fazong Wu, Xihui Ying, Jiansong Ji

**Affiliations:** ^1^ Department of Interventional Radiology, The Fifth Affiliated Hospital of Wenzhou Medical University, Zhejiang University Lishui Hospital, Lishui, Zhejiang 323000, China; ^2^ Department of Radiology, The Fifth Affiliated Hospital of Wenzhou Medical University, Zhejiang University Lishui Hospital, Lishui, Zhejiang 323000, China

**Keywords:** arsenic trioxide, shikonin, ROS, endoplasmic reticulum stress, hepatocellular carcinoma

## Abstract

Hepatocellular carcinoma (HCC) is a highly lethal malignancy mostly because of metastasis, recurrence and acquired resistance to conventional chemotherapy. Arsenic trioxide (ATO) is successfully used to treat hematological malignancies, and has been proven to trigger apoptosis in HCC cells. However, the phase II trial evaluating the efficacy and toxicity of ATO in patients with HCC showed that single-agent ATO is poorly active against HCC. Therefore, it is of great importance to develop effective chemosensitization agents to ATO. The aim of the present study was to determine whether shikonin (SHI), a natural product from the root of lithospermum erythrorhizon, could synergistically enhance the anti-HCC efficacy of ATO both *in vitro* and *in vivo*. We found that the combination of SHI and ATO exhibited synergistic anticancer efficacy and achieved greater selectivity between cancer cells and normal cells. By inducing intracellular oxidative stress, SHI potentiated ATO-induced DNA damage, followed by increased activation of endoplasmic reticulum stress. In addition, inhibition of ROS reversed the apoptosis induced by SHI and ATO, and recovered the activation of endoplasmic reticulum stress, which revealed the vital role of ROS in the synergism. Moreover, HepG2 xenograft tumor growth in nude mice was more effectively inhibited by combined treatment with SHI and ATO. These data suggest that the combination of SHI with ATO presents a promising therapeutic approach for the treatment of HCC.

## INTRODUCTION

Hepatocellular carcinoma (HCC) is the most common liver malignancy worldwide, and its incidence is increasing [1]. Despite the ongoing improvement in management of HCC, little success has been achieved in ameliorating the situation of this disease [2]. Currently, there are few effective therapies partly because the molecular- and cell-based mechanisms that contribute to the pathogenesis of this cancer type are little understood. Systemic chemotherapy is one of the few treatment choices for those unresectable HCC patients [3]. Sorafenib, an oral multikinase inhibitor, is the current standard for the treatment of unresectable HCC [4]. However, the clinical efficacy of sorafenib is modest, and some patients are intolerant [5]. Thus, more effective therapeutic strategies for advanced HCC are needed.

Arsenic trioxide (ATO) is an effective anticancer drug used in patients with relapsed acute promyelocytic leukemia (APL), and is intensively investigated for treatment of other tumors [6, 7]. However, ATO is much less effective against solid tumors such as HCC, colon carcinoma and gastric cancer. Moreover, chronic exposure to low-concentrations of ATO (1 mg/kg) can enhance tumor growth and metastasis, but cannot induce tumor cell death in a mouse model of HCC [8]. The phase II study evaluating the clinical and biologic effects of ATO in patients with HCC indicated that ATO is poorly effective [9]. Recently, intensive combination chemotherapy has been considered for the treatment of cancer, and found that combination chemotherapy had a better effects than those treated with single-agent therapy [10, 11]. Hence, searching of effective agents that could increase the effect of ATO and reduce side effects is very important.

Reactive oxygen species (ROS) are chemically reactive molecules that have important functions in mammalian cells. Mounting research reveals that, compared with their normal counterparts, cancer cells have increased levels of ROS and are under oxidative stress due to an imbalanced redox status [12, 13]. Consequently, cancer cells are more sensitive to agents that further increase ROS levels and oxidative stress [14, 15]. Recent studies have revealed that ATO-induced apoptosis in cancer cells is partially mediated through ROS production [16, 17]. Therefore, chemical agents which can alter the antioxidant defenses and induce oxidative stress have been proposed as potential candidates for enhancing the efficacy of ATO in cancer treatment.

Shikonin (SHI), a natural product isolated from the *lithospermum erythrorhizon*, has been identified as a multifunctional bioactive natural product. Numerous studies have shown that SHI induces apoptosis in cancer cells *in vitro* and *in vivo* [18, 19]. These anti-tumor activities are attributed to the regulation of cell cycle, apoptosis and invasion of tumor cells. A recent study showed that SHI is able to induce apoptosis in human promyelocytic leukemia HL-60 cells through generation of ROS [20]. SHI treatment significantly induced ROS production in cancer cells but not in normal cells, which may underlie SHI's selective cancer killing ability [20, 21]. Moreover, shikonin is able to induce apoptosis in HCC cells via increasing ROS production [22]. In the present study, we intended to identify the hypothesis that SHI, a natural inducer of ROS, could enhance the cytotoxicity of ATO in HCC cells. In addition, we investigated the possible mechanisms underlying cell death induced by combined treatment with SHI and ATO in HCC cells.

## RESULTS

### SHI potentiates the proliferation inhibition effect of ATO in HCC cells *in vitro*

**Figure 1:**
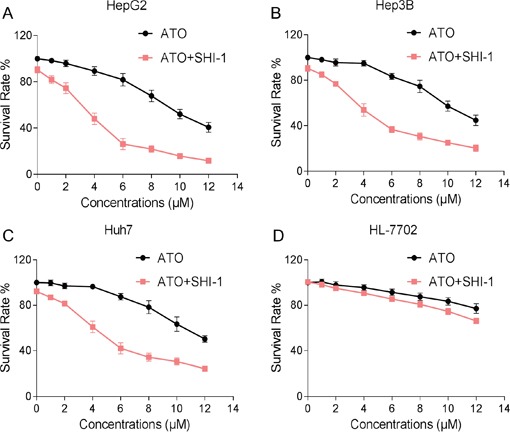
SHI potentiates the proliferation inhibition effect of ATO in HCC cells *in vitro* **A-C.** SHI enhances ATO-induced growth inhibition in HCC cells. Briefly, cells were treated with SHI and ATO for 24 h, cell viability was determined by MTT assay. Assays were performed in triplicate. **D.** SHI and ATO combined treatment showed less cytotoxicity toward HL-7702 normal human liver cells. HL-7702 cells were treated with SHI and ATO for 24 h, cell viability was determined by MTT assay. Assays were performed in triplicate.

We first determined the effect of combination therapy of SHI and ATO on viability of HCC cells. As show in Figure 1A, SHI treatment alone did not significantly decreased the viability of HepG2 cells. However, combination of SHI and ATO exhibited better cytotoxicity on HepG2, Hep3B and Huh7 cells than the use of a single agent (Figure 1A-1C). Moreover, combination of SHI and ATO displayed less cytotoxicity toward HL-7702 normal human liver cells, suggesting the selectivity of SHI in combination ATO (Figure 1D). These results suggested that SHI can sensitize HCC cell lines to the cell killing effects of ATO.

### SHI potentiates the apoptotic effects of ATO in HCC cells

**Figure 2:**
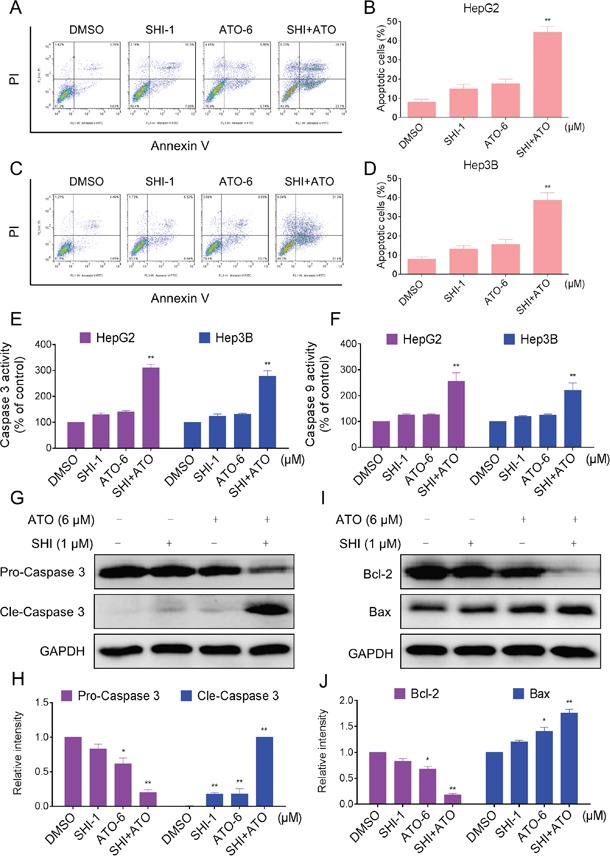
SHI potentiates the apoptosis-inducing effect of ATO in HCC cells *in vitro* **A.** SHI enhances ATO-induced apoptosis in HepG2 cells. Cells after treatment for 24 h were collected and stained with Annexin V and PI. Cell apoptosis rate was analyzed by flow cytometer. Similar results were obtained in three independent experiments. **B.** The percentage of apoptotic cells in the treatment groups was calculated. **C.** SHI enhances ATO-induced apoptosis in Huh7 cells. **D.** The percentage of apoptotic cells in the treatment groups was calculated. **E-F.** Cells after treatment for 20 h were lysed and cell proteins were used to determine caspase 3 and caspase 9 activities with a fluorescence assay kit using specific substrates. **G-J.** Western blot analysis of expression levels of caspases, Bcl-2 and Bax in HepG2 cells. Cells after treatment for 20 h were collected and lysed, cell proteins (50 μg) were separated by SDS-PAGE and immunoblotted with specific primary antibodies. All images shown here are representative of three independent experiments with similar results. **P < 0.01 versus control group.

To determine whether the growth inhibition of HCC cells by combined treatment was caused by apoptosis, we then evaluated the pro-apoptotic effect of SHI, ATO and ATO+SHI on HepG2 and Hep3B cells. As shown in Figure 2A-2D, combined treatment with SHI and ATO resulted in a significant increase in the number of apoptotic cells compared with SHI or ATO alone. We confirmed these findings by caspase acitivity assay and found that SHI markedly increased ATO-induced caspase 3 and caspase 9 activation, suggesting the activation of apoptosis pathway (Figure 2E-2F). The levels of apoptosis-related proteins were also examined by western blotting analysis. As shown in Figure 2G-2J, SHI and ATO cotreatment markedly reduced the levels of pro-caspase 3 and anti-apoptotic protein Bcl-2, and increased the levels of cle-caspase 3 and Bax. Taken together, our findings indicated that combined treatment reduced HCC cells growth through induction of apoptosis.

### ER stress was involved in the SHI and ATO combined treatment-induced apoptosis *in vitro*

**Figure 3:**
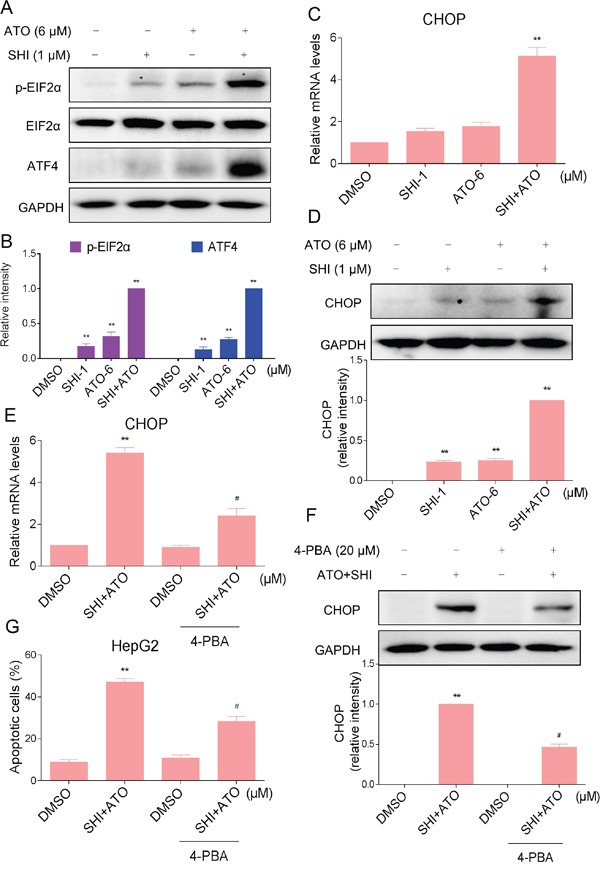
ER stress was involved in the SHI and ATO combined treatment-induced apoptosis *in vitro* **A-B.** Combined treatment increased the expression of p-EIF2α and ATF4 proteins. HepG2 cells were incubated with SHI or/and ATO for 6 h and then the protein levels of p-EIF2α and ATF4 were analyzed by western blotting. A set of representative results is shown from three independent experiments. **C.** Combined treatment increased CHOP mRNA levels in HepG2 cells. HepG2 cells were treated with SHI or/and ATO for 6 h. The mRNA expression of CHOP was analyzed by qRT-PCR. **D.** Combined treatment increased CHOP protein levels in HepG2 cells. HepG2 cells were incubated with SHI or/and ATO for 12 h and then the protein levels of CHOP was analyzed by western blotting. **E-G.** 4-PBA could partially attenuated combined treatment-induced HepG2 cells apoptosis. (E) The mRNA expression of CHOP was analyzed by qRT-PCR. (F) The protein expression of CHOP was analyzed by western blotting. (G) Cell apoptosis rate was analyzed by flow cytometer. **P < 0.01 versus control group, ^#^P < 0.05 versus combined treatment group.

Studies have shown that ER stress has been implicated in ATO-induced apoptosis [23, 24]. Besides, SHI could selectively kill cancer cells by activating ER stress [25]. Therefore, we suspected that activation of ER stress contributes to HCC cell apoptosis by combined treatment. We examined the expression of ER stress-related proteins including eukaryotic initiation factor 2α (p-EIF2α) and activating transcription factor-4 (ATF4) in combined treated HCC cells. As shown in Figure 3A-3B, treatment of cells with SHI (1 μM) and ATO (6 μM) alone both slightly increased the levels of p-EIF2α and ATF4, but combined treatment with SHI and ATO significantly increased the levels of p-EIF2α and ATF4. Meanwhile, we found that combined treatment resulted in significant increases in the mRNA and protein levels of CHOP (Figure 3C-3D). We further investigated whether ER stress was involved in the anti-tumor effects of combined treatment. As shown in Figure 3E-3F, 4-PBA pre-treatment reduced combined treatment-induced CHOP mRNA and protein levels. Meanwhile, we found that 4-PBA pre-treatment markedly decreased combined treatment-induced apoptosis in HepG2 cells (Figure 3G). These results suggested that ER stress was involved in combined treatment-induced HCC cell apoptosis.

### SHI potentiates ATO-induced apoptosis by ROS accumulation

**Figure 4:**
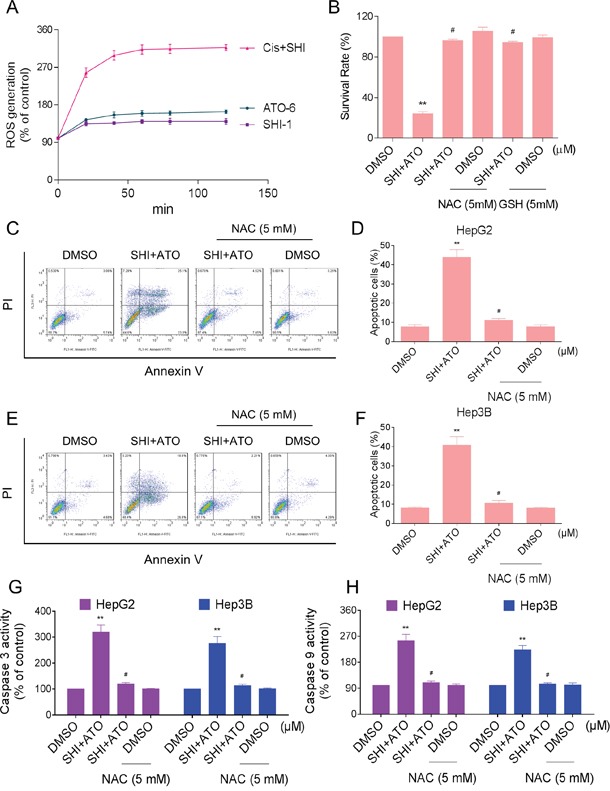
SHI potentiates ATO-induced apoptosis by ROS Accumulation **A.** Intracellular ROS generation induced by SHI and/or ATO was measured by staining with DCFH-DA (10 μM) and flow cytometer. **B.** HepG2 cells were pre-incubated with or without 5 mM NAC or GSH for 2 h before combined treatment. Cell viability was determined by MTT assay. **C-F.** NAC addition protects HCC cells from combined treatment-induced apoptosis. Percentage of cell apoptosis was determined by Annexin-V/PI staining and flow cytometer. **G-H.** NAC addition reverses combined treatment-induced activation of caspase 3 and caspase 9. **P < 0.01 versus control group, ^#^P < 0.05 versus combined treatment group.

ROS promote cell survival and growth by regulating cell signaling cascades [26, 27]. However, over production of ROS may lead to oxidative stress and cell apoptosis [28, 29]. SHI has been found to induce the accumulation of ROS and oxidative stress in cancer cells [20, 30]. Based on these findings, we investigated whether intracellular ROS generation was involved in the anti-tumor effects of combined treatment. As shown in Figure 4A, treatment of cells with SHI (1 μM) and ATO (6 μM) alone both slightly increased intracellular ROS levels as detected by increased DCF levels. However, treatment of HepG2 cells with SHI and ATO significantly increased intracellular ROS levels. This increase was evident as early as 20 min following exposure to SHI and ATO and remained elevated for 2 hours. To determine whether combined treatment-induced ROS participates in mediating cell apoptosis, we utilized N-acetyl cysteine (NAC) and L-glutathione reduced (GSH). Our results showed that pre-treatment of HepG2 cells with NAC and GSH significantly reduced the inhibition of cell proliferation induced by combined treatment (Figure 4B). Furthermore, NAC markedly reversed combined treatment-induced apoptosis in HepG2 and Hep3B cells (Figure 4C-4F). Meanwhile, the activation of caspase 3 and caspase 9 were also reversed (Figure 4G-4H).

**Figure 5:**
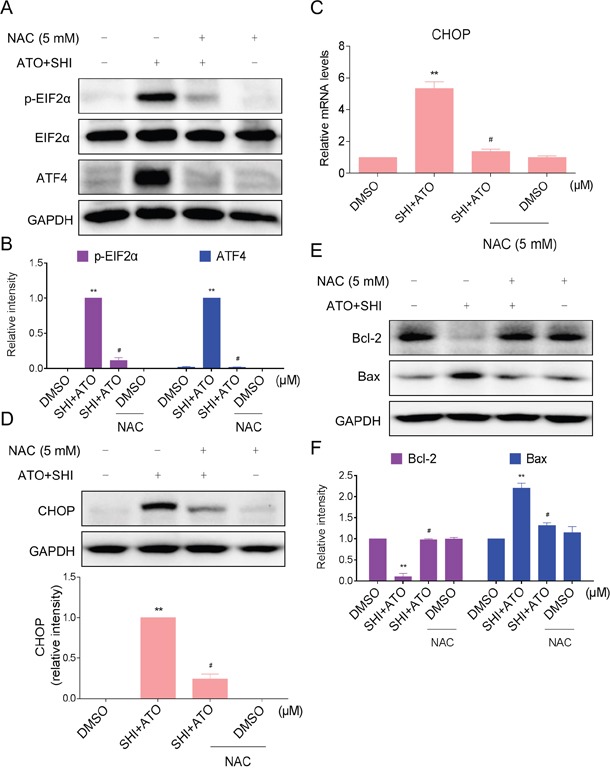
Blocking of ROS generation reversed the activation of ER stress induced by combined treatment **A-B.** HepG2 cells were pre-incubated with or without 5 mM NAC for 2 h before combined treatment. Cell lysates were subjected to western blotting to analyze the expression of p-EIF2α and ATF4. **C.** HepG2 cells were pre-incubated with or without 5 mM NAC for 2 h before combined treatment. The mRNA expression of CHOP was analyzed by qRT-PCR. **D.** HepG2 cells were pre-incubated with or without 5 mM NAC for 2 h before combined treatment. The protein expression of CHOP was analyzed by western blotting. **E-F.** The protein levels of Bcl-2 and Bax were analyzed by western blotting. **P < 0.01 versus control group, ^#^P < 0.05 versus combined treatment group.

Furthermore, we detected the reverse effect of NAC on combined treatment-induced ER stress in HepG2 cells. As shown in Figure 5A-5B, pre-treatment with NAC markedly reversed the overexpression of p-EIF2α and ATF4 in combined treatment-treated HepG2 cells. In addition, NAC pre-treatment significantly reversed combined treatment-induced increase in CHOP mRNA and protein levels (Figure 5C-5D). Meanwhile, the increase of Bax and the reduction of Bcl-2 induced by combined treatment were both blocked by NAC pre-treatment (Figure 5E-5F). These findings indicated that ROS might be the upstream mediator for SHI to increase the apoptosis-inducing ability of ATO in HCC cells.

### SHI increases the anticancer activity of ATO against HCC cells *in vivo*

**Figure 6:**
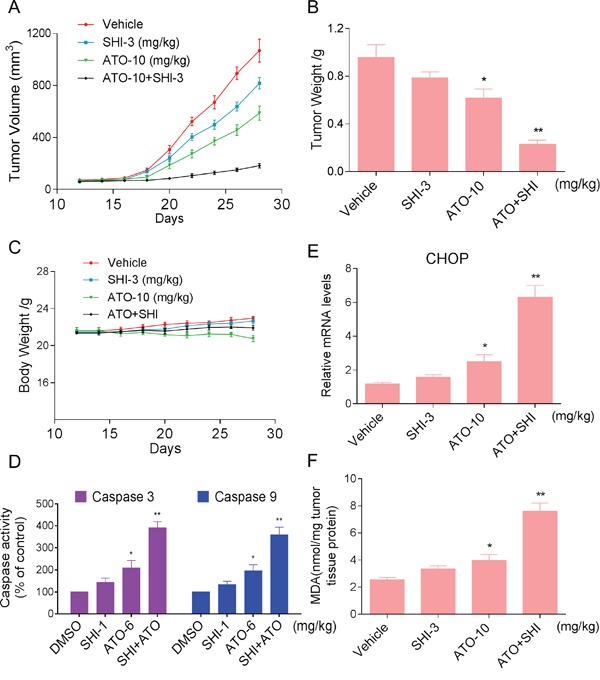
SHI increases the anticancer activity of ATO against HCC cells *in vivo* Combined treatment inhibits tumor volume **A.** and tumor weight **B.** of HepG2 xenografts in nude mice, but do not affect body weight **C.** of mice. **D.** Combined treatment increased caspase 3 and caspase 9 activities *in vivo*. **E.** The levels of CHOP mRNA in the tumor tissues. **F.** The levels of MDA in the tumor tissues. *P < 0.05 versus control group, **P < 0.01 versus control group.

Our last objective was to confirm our *in vitro* findings in a HCC cancer xenograft model. We injected HepG2 cells in athymic nu/nu mice. When the tumors grew to about 100 mm^3^, mice were treated with indicated compounds. As shown in Figure 6A, treatment of ATO alone inhibited HCC cancer cell growth in mice. However, combined treatment with SHI and ATO markedly reduced HepG2 tumor volume and weight compared to the vehicle-treated group. Importantly, there was no significant difference in body weight among the vehicle and combined-treated groups (Figure 6A-6C). Mechanistically, we found that combined treatment showed stronger ability in activating caspases activities in tumor tissues (Figure 6D). Besides, combined treatment increased the expression of CHOP mRNA, indicating that combined treatment-induced apoptosis in HepG2 cells is associated with activation of ER-stress *in vivo* (Figure 6E). Moreover, our results showed that combined treatment with SHI and ATO markedly increased the levels of MDA (Figure 6F). These findings indicated that that SHI can synergistically enhance ATO-induced tumor growth inhibition *in vivo* by inducing ROS accumulation.

## DISCUSSION

Hepatocellular carcinoma (HCC) continues to be a global health problem. Sorafenib, an oral multikinase inhibitor with antiproliferative and antiangiogenic effects, currently sets the new standard for unresectable HCC. However, the objective tumor response rates are usually quite low [5]. Arsenic trioxide is an effective anticancer drug used in patients with relapsed acute promyelocytic leukemia, and is investigated for the treatment of unresectable HCC. However, the phase II study of ATO showed that single-agent ATO is not active against advanced HCC [9]. Therefore, the combination of ATO with a sensitizer that can enhance ATO-mediated effects may be necessary for the treatment of HCC [31]. In the present study, we investigated the effect of SHI potentiates the cytotoxic effects of ATO in HCC cells. We found that combination of SHI and ATO is more effective than single agents in cancer cell lines. Furthermore, we found that SHI synergizes ATO-mediated apoptosis by activating ROS-dependent ER stress pathway.

Over the years, apoptosis has received much attention in emerging as the major mechanism by which anti-tumor agents act to eliminate tumor cells. Apoptotic cells exhibit typical apoptotic features such as caspase activation, apoptotic body formation and DNA fragmentation [32]. We found that combination of SHI and ATO exerts a significant proapoptotic effect on HCC cells. Recent evidence showed that ATO induces apoptosis of cancer cells through activating ER stress pathway [23]. In the present study, we found that combined treatment with SHI and ATO significantly activated the ER stress pathway in HCC cells as evidenced by increased levels of p-EIF2α and ATF4. Meanwhile, ER stress marker CHOP was significantly increased in HCC cells treated with SHI and ATO. Moreover, our findings demonstrated that blocking ER stress by 4-PBA significantly decreased combined treatment-induced apoptosis in HCC cells. Taken together, these findings suggested that ER stress was involved in combined treatment-induced apoptosis in HCC cells. Moreover, our findings indicated that targeting ER stress is a useful anti-cancer strategy and dissecting out these interactions in HCC cells will likely provide new targets for therapy.

Compared with normal cells, many types of cancer cell have increased levels of ROS. Therefore, it might be possible to selectively kill cancer cells by pharmacological ROS insults [14, 29]. In the previous studies, SHI exhibits potent anti-tumor activities via ROS-mediated apoptosis [20, 30]. Our findings showed that SHI markedly increased ATO-induced apoptosis by promoting ROS production, and blockage of ROS production by NAC or GSH totally reversed the cell death induced by combined treatment with SHI and ATO in HepG2 cells. In addition, combined treatment-induced activation of ER stress and mitochondrial pathway were almost totally reversed by NAC, suggesting that ROS production is the upstream regulator. Previous study have shown that SHI interacts with thioredoxin reductase 1 to induce ROS in HL-60 cells [20]. Therefore, it is possible that SHI synergizes ATO-mediated apoptosis in HCC cells by inhibiting thioredoxin reductase 1 activity. Elucidating these mechanisms is a pressing issue and a focus of future studies.

In conclusion, we investigated the synergistic anti-tumor effects of combined SHI and ATO in HCC cells and the potential underlying mechanisms. We found that SHI significantly enhanced ATO-induced HCC cells killing *in vitro* and *in vivo*, and demonstrated that combined treatment induces apoptotic cell death through increased production of ROS and activation of ER stress pathway. These results indicated that the combination of SHI and ATO possesses great potential as a promising strategy for the treatment of HCC. In addition, our findings indicated that ROS production and ER stress could be viewed as a potential strategy for the development of new anti-tumor agents.

## MATERIALS AND METHODS

### Cell culture and reagents

Arsenic trioxide (ATO), Shikonin (SHI), 4-phenylbutyric acid (4-PBA), N-acetylcysteine (NAC) and L-Glutathione reduced (GSH) were purchased from Sigma (St. Louis, MO). Human HCC cell lines HepG2, Hep3B, Huh7, and normal human liver cell line HL-7702 were purchased from the Institute of Biochemistry and Cell Biology, Chinese Academy of Sciences. The cells were cultured in RPMI-1640 medium containing 10% Fetal Bovine Serum, 100 units/ml penicillin, and 100 μg/ml streptomycin at 37°C in a humidified atmosphere with 5% CO_2_. FITC Annexin V and Propidium Iodide (PI) were purchased from BD Pharmingen (Franklin Lakes, NJ). Antibodies against ATF4, p-EIF2α, EIF2α, and CHOP were purchased from Cell Signaling Technology (Danvers, MA). Antibodies against Bcl-2, Bax, caspase-3 p30/17, and GAPDH were purchased from Santa Cruz Biotechnology (Santa Cruz, CA).

### Cell viability assay

Cells were seeded into 96-well plates at a density of 6×10^3^ per well and allowed to grow overnight. ATO was dissolved in DMSO and diluted with 1640 medium to final concentrations of 2, 4, 6, 8, 10 and 12 μM. The cells were incubated with ATO or with a combination of ATO and SHI (1 μM) for 24 h before the MTT assay.

### Cell apoptosis analysis

Apoptosis was measured with FITC conjugated Annexin V and Propidium Iodide (PI) in binding buffer using a FACSCalibur flow cytometer (BD Biosciences, CA). Briefly, cells were washed twice with cold PBS, and then resuspended in 100 μL 1× binding buffer. 5 μL of annexin V-FITC and 5 μL of PI were added. Thereafter, these cells were incubated for 15 min at room temperature in the dark, and then 400 μL of binding buffer was added to each tube. The cells were analyzed by FACSCalibur flow cytometer.

### Western blot analysis

Cells or tumor tissues were homogenized in protein lysate buffer, and debris was removed by centrifugation at 12,000 g for 12 min at 4°C. The protein concentrations in all samples were determined by using the Bradford protein assay Kit (Bio-Rad, Hercules, CA). Protein samples were electrophoresed and then transferred to PVDF membrane. The membrane was blocked for 1 h at room temperature with fresh 5% nonfat milk in TBST, and then incubated with primary antibody overnight at 4°C and secondary antibody for one hour at room temperature. The immunoreactive bands were visualized by using ECL Kit (Bio-Rad, Hercules, CA). The density of the immunoreactive bands was analyzed using Image J computer software.

### Quantitative RT-PCR

Cells were homogenized in TRIzol reagent (Invitrogen, Carlsbad, CA) for extraction of RNA according to each manufacturer's protocol. Both reverse transcription and quantitative PCR were carried out using a two-step M-MLV Platinum SYBR Green qPCR SuperMix-UDG Kit (Invitrogen, Carlsbad, CA). Eppendorf Mastercycler eprealplex detection system (Eppendorf, Hamburg, Germany) was used for q-PCR analysis. The following gene-specific primer pairs were used: CHOP: (F) 5′-atggcagctgagtcattgcctttc-3′, (R) 5′-agaagcagggtcaagagtggtgaa-3′. β-actin: (F) 5′-ttcctgggcatg gagtcct-3′, (R) 5′-aggaggagcaatgatcttgatc-3′. Gene expressions were analyzed with the comparative threshold cycle (Ct) method after normalizing to the housekeeping gene β-actin.

### Measurement of reactive oxygen species

Cellular ROS levels were measured by flow cytometer. Briefly, cells were plated on 60-mm dishes and allowed to grow overnight. After treatment, cells were stained with 10 μM DCFH-DA (Beyotime Biotech, China) at 37°C for 30 min. Cells were collected and the fluorescence was analyzed using a FACSCalibur flow cytometer.

### Determination of caspase 3/9 activity

Caspase 3/9 activity in cell lysates was determined using a Caspase 3/9 activity Kit (Beyotime Biotech, China) according to the manufacturer's protocol. The caspase 3/9 activity was normalized by the protein concentration of the corresponding cell lysate and expressed as percentage of treated cells to that of control.

### *In vivo* antitumor study

All surgical procedures and care administered to the animals were in accordance with institutional animal ethic guidelines. Animals were housed at a constant room temperature with a 12 h light/12 h dark cycle and fed a standard rodent diet and water. Tumors were established by subcutaneous injection of 5 × 10^6^ HepG2 tumor cells into the flanks of mice. When tumors reached a volume of about 100 mm^3^, the mice were randomly assigned to 4 groups (each group had seven mice): control, SHI, ATO, ATO + SHI. Mice were treated by intraperitoneal (i.p.) injection of 10 mg/kg ATO once per day, or by i.p. injection of 3 mg/kg SHI once per day, or with a combination of ATO and SHI according to the same schedules. The tumor volumes were determined by measuring length (l) and width (w) and calculating volume (V = 0.5 × l × w^2^) at the indicated time points. At the end of treatment, the animals were sacrificed, and the tumors were removed and weighed.

### Statistical analysis

All experiments were assayed in triplicate (n = 3). Data are expressed as means ± SEM. All statistical analyses were performed using GraphPad Pro. Prism 5.0. Student's t-test and two-way ANOVA were employed to analyze the differences between sets of data. A *p* value <0.05 was considered statistically significant.
